# Hyperreflective Foci in the Ectopic Inner Foveal Layer of Advanced Idiopathic Epiretinal Membranes

**DOI:** 10.1167/iovs.66.9.14

**Published:** 2025-07-07

**Authors:** Nicola Valsecchi, Maurizio Mete, Giulio Rapezzi, Matteo Elifani, Giulia Folgaria, Alessandro Finzi, Antonio Moramarco, Luigi Fontana

**Affiliations:** 1 Ophthalmology Unit, Dipartimento di Scienze Mediche e Chirurgiche, Alma Mater Studiorum University of Bologna, Bologna, Italy; 2 IRCCS Azienda Ospedaliero-Universitaria di Bologna, Bologna, Italy

**Keywords:** hyperreflective foci, epiretinal membrane, OCT, biomarker, gliosis

## Abstract

**Purpose:**

To investigate the presence of hyperreflective foci (HF) in the ectopic inner foveal layer (EIFL) of eyes with advanced idiopathic epiretinal membranes (iERMs) before and after pars plana vitrectomy (PPV) with ERM peeling.

**Methods:**

A retrospective analysis was conducted on clinical records and spectral-domain optical coherence tomography (SD-OCT) scans from consecutive patients who underwent PPV with ERM peeling. Stage 3 and stage 4 iERMs were included. HF were defined as dot-like lesions (<30 µm) with reflectivity similar to that of the retinal nerve fiber layer, lacking backshadowing, located in the EIFL thickness within 1500 µm of the fovea. Patients were followed up to twelve months after surgery.

**Results:**

A total of 47 eyes were included: 10 stage 4 iERM and 37 stage 3 iERM. HF in EIFLs were observed in 74.5% of cases, with higher prevalence in stage 4 than stage 3 (100% vs. 67.6%; *P* = 0.035). At baseline, an increase in HF count was associated with greater EIFL thickness (*r* = 0.497, *P* < 0.001). At 7.8 ± 1.8 months post-surgery, HF were found in 59.6% of eyes. Eyes with persistent HF had greater postoperative EIFL thickness than those without (143 ± 60.4 vs. 103.7 ± 57.3; *P* = 0.036). In multivariate linear regression, the presence of HF at baseline (β = 0.373, *P* = 0.048) was significantly associated with worse postoperative visual acuity.

**Conclusions:**

HF in the EIFL were more prevalent in advanced iERM and correlated with increased EIFL thickness. Their presence at baseline was associated with worse postoperative visual acuity.

Idiopathic epiretinal membrane (iERM) is a retinal disease characterized by the formation of a fibrous cellular membrane on the retinal surface in the macular area. The prevalence of iERM ranges from 2.2% to 28.9%, with the risk of its development increasing with advancing age.^1^^,^^2^ iERM can be asymptomatic; however, when it affects the foveal region, it typically leads to a range of symptoms, including blurred vision and metamorphopsia, potentially progressing to a substantial decrease in best-corrected visual acuity (BCVA).^3^

Although the exact mechanisms underlying iERM remain incompletely understood, it is believed to be associated with posterior vitreous detachment (PVD), which causes damage to the internal limiting membrane (ILM).^4^ This damage subsequently activates and induces the migration of retinal pigment epithelium (RPE) cells, hyalocytes, and glial cells toward the ILM.^5^ Also, anomalous PVD with vitreoschisis could be implicated in the pathogenesis of iERM formation.^6^^,^^7^ The remaining vitreous cortex on the retinal surface may serve as a scaffold, promoting the migration and proliferation of inflammation-related cells, thus facilitating the formation of the pathological epiretinal membrane.^8^ Several cytokines and inflammatory molecules have been identified as playing a role in the development of this pathology.^9^

Govetto et al.^10^ proposed an optical coherence tomography (OCT)-based classification that helps to predict disease progression and outcomes following pars plana vitrectomy (PPV). The persistent traction exerted by the ERM has been found to induce a reorganization of the inner retina, leading to the development of ectopic inner foveal layers (EIFLs). Also, animal studies have shown that tractional stress can promote Müller cell proliferation and gliosis, contributing to formation of the EIFL.^11^^,^^12^ This retinal tissue plays a crucial role in advanced iERM, as it can significantly impact visual acuity both before and after surgery.^13^^,^^14^

Numerous studies have explored the presence of hyperreflective foci (HF) in various retinal conditions, such as diabetic macular edema, branch retinal vein occlusions, and age-related macular degeneration.^15^ These HF are considered a unique inflammatory phenotype, composed of microglial cells, perivascular macrophages, monocyte-derived macrophages, and a subset of vitreous-resident cells.^16^ In a recent investigation, Zhao et al.^17^ observed HF at the vitreous–retinal interface in cases of idiopathic iERM using en face OCT. They suggested that these foci might represent inflammatory cells involved in the pathogenesis of iERM. Because inflammatory processes are also implicated in the formation of EIFLs, HF could also be present within the EIFL and potentially play a role in the progression of advanced iERM. Therefore, the primary objective of this study was to evaluate the presence of HF within the EIFL thickness before and after surgery and to correlate these changes with various OCT parameters. In addition, we investigated the potential role of HF as a predictor of postoperative visual recovery.

## Materials and Methods

### Study Population

This was a retrospective, observational study based on a review of consecutive patients’ clinical records affected by stages 3 and 4 iERM^10^ who underwent PPV and ERM peeling at the Unit of Ophthalmology, University of Bologna, between November 2021 and December 2024. We excluded patients with stage 1 and 2 iERM, as these patients do not present EIFLs. All patients included in the study underwent baseline assessments, as well as postoperative follow-up examinations at 1, 3, 6, and 12 months. The exclusion criteria were significant media opacities (other than cataracts), other macular conditions (such as intermediate or late-stage age-related macular degeneration or diabetic macular edema), poor-quality imaging, any prior ocular surgery (except cataract surgery), and an axial length greater than 26 mm. All procedures adhered to the tenets of the Declaration of Helsinki, and all participants provided written informed consent. This study was approved by the Ethics Committee of Bologna, Italy (Cod CE: 53/2025/Oss/AOUBo).

### Ophthalmological Assessment

At baseline and each follow-up visit, a comprehensive ophthalmological examination was conducted, which included BCVA measurement, slit-lamp biomicroscopy, intraocular pressure assessment, dilated fundus examination with a 90-diopter (D) indirect lens, and spectral-domain OCT (SD-OCT) imaging (SPECTRALIS HRA-OCT; Heidelberg Engineering, Heidelberg, Germany). Axial length was also measured at baseline (IOLMaster 700; Carl Zeiss Meditec, Jena, Germany). BCVA was assessed using a Snellen chart and converted to the logarithm of the minimum angle of resolution (logMAR) for statistical analysis.

### OCT Image Acquisition

All subjects underwent the same SD-OCT protocol, including a dense 20° × 15° raster scan with 145 B-scans spaced 30 µm apart, along with two vertical and horizontal single scans centered on the fovea (automatic real-time values of at least 14 or higher). ERM was defined as a distinct, irregular, hyperreflective line anterior to the inner retinal surface, associated with wrinkling of the underlying retina. Two masked graders (NV, ME) independently assessed OCT scans for ERM classification, including stages 3 and 4 iERM. In cases of uncertainty, a third grader (MM) made the final decision. The thickness of the EIFL in the foveal region was measured manually using the caliper function of the Heidelberg instrument, as previously described.^13^ All images were adjusted to a 1:1-mm aspect ratio. Central macular thickness (CMT) was manually measured as the distance between the ILM and the anterior border of the RPE–Bruch's membrane complex at the fovea. Subfoveal choroidal thickness (SFCT) was measured manually as the vertical distance between the hyperreflective line of the Bruch’s membrane and the inner scleral surface using the caliper tool of the image analysis software. Disruption of the inner segment ellipsoid zone (EZ) was defined as a discontinuous ellipsoid band in the foveal region. The central bouquet alteration was defined based on previous publications and included any of the following abnormalities within the central 100 µm of the fovea at the level of the EZ band: the cotton ball sign, a central pocket of subretinal fluid, or a central vitelliform lesion.^13^^,^^18^ Additionally, the presence of hyporeflective intraretinal cystoid spaces in the context of ERMs was evaluated, and, in some patients with intraretinal cystoid spaces, fluorescein angiography (FA) was performed using the Heidelberg SPECTRALIS SD-OCT. Microcystoid macular edema (MME) was distinguished from cystoid macular edema (CME) based on a prior publication.^19^ Briefly, the OCT differentiation between MME and CME was based on the location of cystoid spaces (restricted to the inner nuclear layer in MME, potentially involving all retinal layers in CME), foveal involvement (absent in MME, typically present in CME), and cystoid space confluence (absent in MME, usually present in CME). Also, CME showed the classic petaloid pattern on FA, whereas MME showed no leakage or mild capillary leakage. All OCT images were qualitatively and quantitatively reviewed by two independent and masked observers (GR, ME), with disagreements resolved by a third observer (NV).

### Assessment of HF in the EIFL

**Figure 1.**
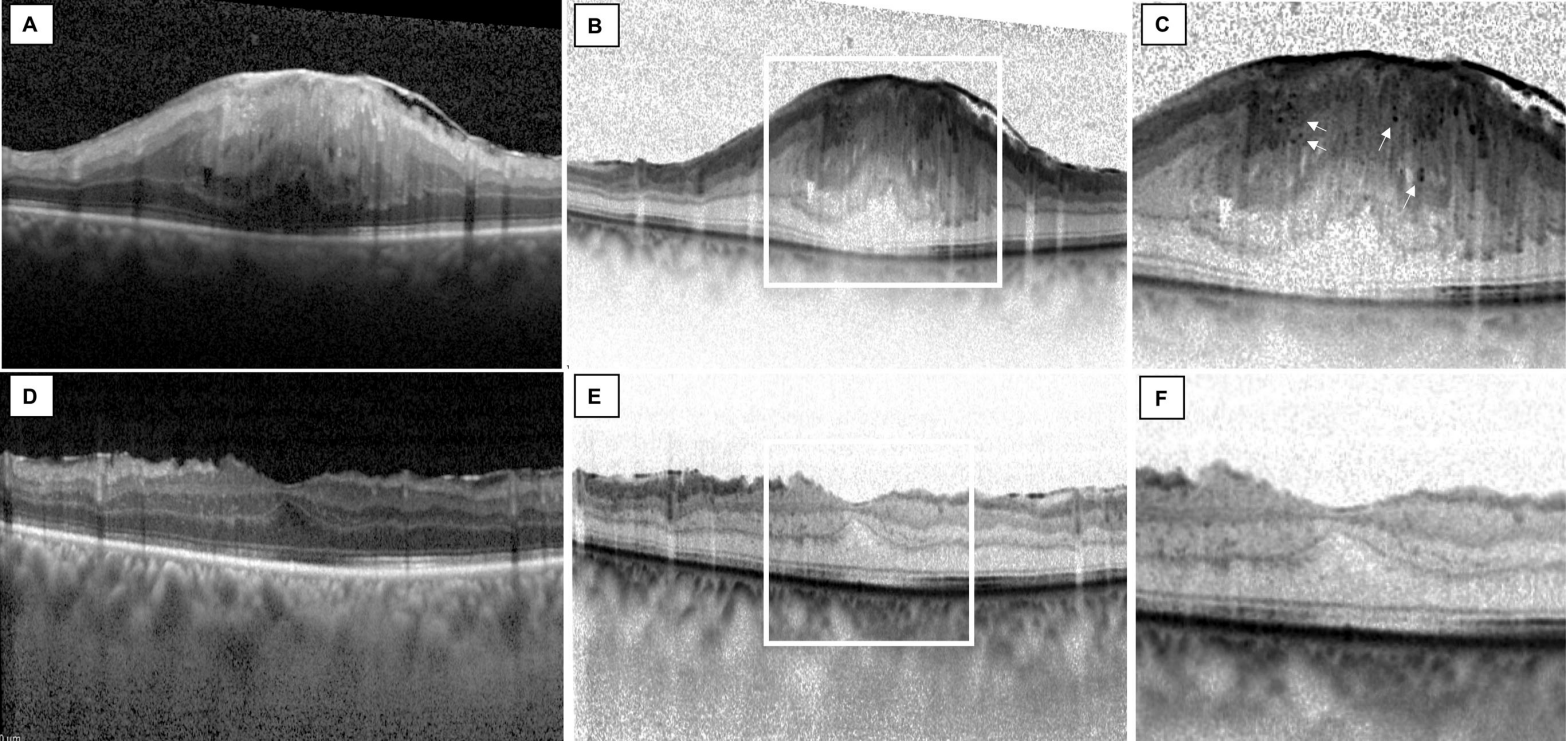
HF in stage 4 iERM. (**A**) OCT vertical B-scan in a 76-year-old male patient with stage 4 iERM in the left eye. (**B**) Black-and-white mode of the same B-scan. (**C**) Magnification of the fovea is shown; note the presence of HF in the EIFL thickness (*white arrows*). (**D**, **E**) Vertical OCT B-scan 6 months after ERM peeling. (**F**) Note the reduction of HF in the central 1500 µm.

The presence of HF was evaluated in all SD-OCT scans obtained at baseline and after surgery. HF were defined as dot-like lesions (>10 µm and <30 µm) with reflectivity similar to the retinal nerve fiber layer, lacking backshadowing, and localized within the EIFL thickness within 1500 µm of the fovea. The 1500 µm of the fovea were assessed using the caliper function of the Heidelberg SPECTRALIS OCT (Fig. 1). Two masked graders (GR, ME) manually counted the HF in the EIFL in OCT scans. The measurements from the first grader (GR) were used for the analysis, whereas the measurements from the second grader (ME) were used to assess the intergrader reliability. Also, HF were classified as “absent,” “few” (<10), “moderate” (10–20), or “numerous” (>20). After surgery, persistence of HF was defined as the presence of foci at follow-up that remained within the same classification category as at baseline. Reduction of HF was defined as a decrease in the number of foci, corresponding to a shift to a lower category on the scale. In case of uncertainty about staging, a third observer (NV) made the final decision on the grading. Furthermore, HF were also qualitatively assessed in the outer nuclear layer (ONL) in the central 1500 µm of the fovea, using the same criteria.

### Surgical Technique

All patients underwent the same surgical technique. General or local anesthesia was chosen according to patient preference and the anesthesiologist's indication. A 25-gauge PPV was performed using the Constellation platform (Alcon, Geneva, Switzerland). Cataract surgery was performed before PPV in phakic eyes. Patients underwent complete vitrectomy and peripheral vitreous shaving, and a posterior hyaloid separation from the retina was obtained if needed. Both the ERM and ILM were removed to the vascular arcades using microforceps (Grieshaber ILM forceps; Alcon). MEMBRANEBLUE-DUAL dye (DORC, Zuidland, The Netherlands) was applied to the retinal surface to improve the visualization of the ERM and ILM during the peeling process. Fluid–air exchange was performed at the end of the procedure. All patients followed a standard postoperative regimen of topical antibiotic and anti-inflammatory medications.

### Statistical Analysis

Demographic and clinical data were summarized using descriptive statistics, including measures of precision and variability, based on the type of variable (categorical or continuous). Normality in the distribution of continuous data was assessed using the Shapiro–Wilk test, and parametric tests were applied for the analysis. Interrater reliability for HF counting was assessed using the absolute agreement model of the intraclass correlation coefficient (ICC). Fisher's exact test was used to examine associations between categorical variables. A *t*-test was employed to compare demographic data, EIFL, CMT, SFCT, and BCVA between stage 3 and stage 4 iERM. Also, a *t*-test was used to compare clinical and OCT parameters between eyes with and without HF in the EIFL and baseline and after surgery. Analysis of variance (ANOVA) was utilized to assess EIFL, CMT, SFCT, and BCVA in relation to the quantity of HF. A paired *t*-test was used to compare EIFL, CMT, SFCT, and BCVA before and after surgery. A multivariate linear regression analysis was performed to investigate the linear relationship between BCVA at the final visit (dependent variable) and a set of predictor variables. *P* < 0.05 was considered statistically significant. All statistical analyses were conducted using SPSS Statistics 26 (IBM, Chicago, IL, USA).

## Results

### Demographic Data

A total of 47 eyes were included in the analysis: 10 eyes (21.8%) with stage 4 iERM and 37 eyes (78.7%) with stage 3 iERM. The mean age of the patients was 72.8 ± 7.4 years, and 25 patients were female (53.2%). Of the eyes included, 28 (59.5%) underwent phacoemulsification and intraocular lens (IOL) implantation before pars plana vitrectomy, whereas the remaining eyes had previously undergone phacoemulsification. The mean BCVA at baseline was 0.54 ± 0.51 (Table 1). The mean follow-up after surgery was 7.8 ± 1.8 months.

**Table 1.** Clinical and OCT Features in Stage 3 and 4 iERM at Baseline

	iERM		
Baseline	Stage 3 (*n* = 37)	Stage 4 (*n* = 10)	*P*
Age, mean ± SD	73.3 ± 7.2	68.7 ± 7.3	0.084
Sex female, *n* (%)	19 (51.3)	6 (60)	0.730
Phacoemulsification, *n* (%)	22 (59.4)	6 (60)	0.949
BCVA (logMAR), mean ± SD	0.46 ± 0.43	0.75 ± 0.31	0.163
CMT (µm), mean ± SD	476.18 ± 53.68	638.10 ± 96.03	**<0.001**
SFCT (µm), mean ± SD	193.68 ± 80.45	278.0 ± 97.20	**0.015**
EIFL thickness (µm), mean ± SD	141.62 ± 53.76	282.20 ± 85.45	**<0.001**
Quantity of HF in EIFL, mean ± SD	6.37 ± 5.79	19.20 ± 10.64	**0.004**
Presence of HF in EIFL, *n* (%)	25 (67.6)	10 (100)	**0.035**
Few	17 (68)	3 (30)	
Moderate	8 (32)	4 (40)	
Numerous	0	3 (30)	
Presence of HF in ONL, *n* (%)	6 (16.2)	4 (40)	0.118
EZ disruption, *n* (%)	3 (8.1)	7 (70)	**<0.001**
Intraretinal cystoid spaces, *n* (%)	14 (37.8)	9 (90)	**0.004**
MME	11 (84.6)	6 (66.7)	
CME	2 (15.4)	3 (33.4)	
Central bouquet alteration, *n* (%)	9 (24.3)	2 (20)	0.570

Significant *P* values are highlighted in bold; *t*-test was used for quantitative variables and Fisher's exact test for categorical variables.

### Baseline OCT Assessment

At baseline, HF within the EIFL thickness were found in 35 eyes (74.5%). Of these, 20 eyes (57.1%) had few HF, 12 eyes (34.3%) had moderate HF, and three eyes (8.6%) had numerous HF. Additionally, 10 eyes (21.3%) showed foveal EZ disruption, 11 eyes (23.4%) exhibited the central bouquet alteration, and 10 eyes (21.3%) showed HF in the ONL. Intraretinal cystoid spaces were observed in 23 eyes (48.9%); 17 eyes (73.9%) showed MME, and six eyes (26.1%) showed CME. In cases of CME, FA was performed to rule out any retinal inflammatory or vascular conditions. The Fisher's exact test showed a significant association between intraretinal cysts and HF in the ONL at baseline (*P* = 0.004). On the other hand, no association was observed when comparing HF in the EIFL and the presence of intraretinal cysts at baseline (*P* = 0.093). Also, no differences in EIFL thickness were observed comparing eyes with and without HF in the ONL (185.5 ± 86.4 vs. 167.7 ± 84.2; P = 0.560). Differences between stage 3 and stage 4 OCT features are shown in Table 2. We observed a significant association between an increase in HF in EIFL and elevated CMT and EIFL (*P* = 0.014) (Table 3). Also, HF in EIFL count showed a significant positive correlation with both CMT (*r* = 0.442, *P* = 0.002) and EIFL thickness (*r* = 0.497, *P* < 0.001). On the other hand, no significant correlation was found between HF in EIFL count and SFCT (*r* = −0.037, *P* = 0.810) or preoperative BCVA (*r* = 0.257, *P* = 0.249). Overall, both reads showed a good level of agreement in counting HF in EIFL (ICC = 0.844; 95% confidence interval [CI], 0.783–0.892).

**Table 2.** Clinical and OCT Features in Eyes With and Without HF in the EIFL at Baseline

Baseline	HF+(*n* = 35)	HF–(n= 12)	*P*
Age (y), mean ± SD	71.7 ± 7.5	74.7 ± 6.2	0.216
Sex female, *n* (%)	21 (60)	4 (33.3)	0.103
Phacoemulsification, *n* (%)	19 (54.3)	9 (75)	0.207
BCVA (logMAR), mean ± SD	0.58 ± 0.45	0.43 ± 0.30	0.452
CMT (µm), mean ± SD	528.34 ± 94.04	459.00 ± 67.05	**0.010**
SFCT (µm), mean ± SD	225.18 ± 107.59	177.33 ± 58.26	**0.045**
EIFL thickness (µm), mean ± SD	187.17 ± 86.81	125. 91 ± 56.88	**0.009**
Presence of HF in ONL, *n* (%)	10 (28.6)	0	**0.035**
EZ disruption, *n* (%)	9 (25.7)	1 (8.3)	**0.199**
Intraretinal cystoid spaces, *n* (%)	20 (57.1)	3 (25)	**0.055**
MME	14 (70)	3 (100)	
CME	6 (30)	0	
Central bouquet alteration, *n* (%)	7 (20)	2 (16.7)	0.285

Significant *P* values are highlighted in bold; *t*-test was used for quantitative variables and Fisher's exact test for categorical variables.

**Table 3.** HF and Clinical and OCT Features

	Mean ± SD				
Baseline	HF Absent	HF Few (<10)	HF Moderate (>10 and <20)	HF Numerous (>20)	*P*
BCVA (logMAR)	0.43 ± 0.30	0.63 ± 0.65	0.44 ± 0.16	0.83 ± 0.45	0.492
CMT thickness (µm)	459 ± 67.05	514.10 ± 80.61	524.16 ± 90.91	640 ± 149.40	**0.014**
SFCT thickness (µm)	177.33 ± 58.26	249.15 ± 120.75	202.54 ± 84.13	156.33 ± 56.76	0.154
EIFL thickness (µm)	125.91 ± 56.88	168.45 ± 80.82	193.50 ± 82.08	286.66 ± 102.06	**0.014**

Significant *P* values are highlighted in bold (ANOVA).

### Postsurgery OCT Assessment

At the end of the follow-up, HF in the EIFL were observed in 28 eyes (59.6%) after surgery. A significant improvement in BCVA (preoperative, 0.54 ± 0.46; postoperative, 0.25 ± 0.56; *P* < 0.001), and a significant reduction in CMT (preoperative, 508.74 ± 95.25; postoperative, 371.93 ± 69.90; *P* = 0.012), SFCT (preoperative, 216.51 ± 101.67; postoperative, 196.02 ± 84.50; *P* < 0.001), EIFL thickness (preoperative, 169.09 ± 84.55; postoperative, 125.62 ± 61.60; *P* = 0.011), and HF count (preoperative, 9.04 ± 8.83; postoperative, 3.91 ± 4.48; *P* < 0.001) were observed. Among the entire cohort of patients, 12 eyes (25.5%) presented intraretinal cystoid spaces (nine MME and three CME), nine eyes (19.1%) showed EZ disruption, six eyes (12.7%) had the central bouquet alteration, and 12 eyes (25.5%) showed HF in the ONL. Among eyes with HF in EIFL at baseline (*n* = 35), we observed a complete resolution of HF after surgery in seven eyes (20%), a reduction in six eyes (17.1%), and persistence of the HF in 22 eyes (62.8%). Eyes with stage 3 iERM presented HF in EIFL in 51.3% (19/37) of the cases after surgery, whereas eyes with stage 4 iERM presented HF in EIFL in 90% (9/10) of the cases after PPV (*P* = 0.028) (Table 4). Fisher’s exact test showed a significant association between the presence of HF in the ONL postsurgery and the presence of intraretinal cysts (*P* = 0.008). However, no difference was observed when comparing HF in the EIFL and the presence of intraretinal cysts after surgery (*P* = 0.340). Also, no differences in EIFL thickness were observed comparing eyes with and without HF in the ONL (158.5 ± 86.4 vs. 112.8 ± 84.2; *P* = 0.056).

**Table 4.** Clinical and OCT Features in Stage 3 and 4 iERM After Surgery

	iERM		
	Stage 3 (*n* = 37)	Stage 4 (*n* = 10)	*P*
BCVA (logMAR), mean ± SD	0.21 ± 0.46	0.23 ± 0.32	0.959
CMT (µm), mean ± SD	361.38 ± 67.89	411.77 ± 64.52	0.052
SFCT (µm), mean ± SD	185.44 ± 79.12	264.57 ± 101.07	0.090
EIFL thickness (µm), mean ± SD	122.08 ± 67.95	139.0 ± 24.90	0.471
Quantity of HF in EIFL, mean ± SD	3.05 ± 3.81	7.00 ± 5.51	**0.012**
HF in EIFL, *n* (%)	19 (51.3)	9 (90)	**0.028**
Few	16 (84.2)	5 (55.5)	
Moderate	3 (15.8)	4 (44.6)	
Numerous	0	0	
Presence of HF in ONL	10 (27.1)	2 (20)	0.441
EZ disruption, *n* (%)	2 (5.4)	7 (70)	**<0.001**
Intraretinal cystoid spaces, number (%)	7 (18.9)	5 (50)	0.060
MME	6 (85.7)	3 (60)	
CME	1 (14.2)	2 (40)	
Central bouquet alteration, *n* (%)	6 (16.2)	0	0.208

Significant *P* values are highlighted in bold; *t*-test was used for quantitative variables and Fisher's exact test for categorical variables.

**Table 5.** Clinical and OCT Features in Eyes With and Without HF in the EIFL After Surgery

	HF+(*n* = 28)	HF−(*n* = 19)	*P*
BCVA (logMAR), mean ± SD	0.33 ± 0.57	0.10 ± 0.20	0.148
CMT (µm), mean ± SD	386.91 ± 70.98	353.00 ± 64.72	0.114
SFCT (µm), mean ± SD	215.42 ± 112.64	184.52 ± 46.72	0.300
EIFL thickness (µm), mean ± SD	143.00 ± 60.37	103.68 ± 57.33	**0.035**
Presence of HF in ONL, *n* (%)	9 (32.1)	3 (15.8)	0.125
EZ disruption, *n* (%)	8 (28.5)	1 (5.2)	**0.043**
Intraretinal cystoid spaces, *n* (%)	9 (32.1)	3 (15.7)	0.179
MME	6 (70)	3 (100)	
CME	3 (30)	0	
Central bouquet alteration, *n* (%)	3 (10.7)	3 (15.7)	0.484

Significant *P* values are highlighted in bold; *t*-test was used for quantitative variables and Fisher's exact test for categorical variables.

**Table 6.** Multivariate Linear Regression Analysis of Clinical and OCT Features Associated With the Presence of HF in Eyes After Surgery

	Beta	95% CI		*P*
Presence of HF in EIFL	0.373	0.003	0.681	**0.048**
Intraretinal cysts	−0.055	−0.376	0.280	0.764
EZ/ELM foveal disruption	0.018	−0.806	0.849	0.189
Central bouquet alteration	−0.031	−0.374	0.309	0.844
Presence of HF in ONL	0.079	−0.352	0.541	0.669
Age	0.610	0.007	0.060	**0.015**
Cataract	0.207	−0.235	0.608	0.369
Baseline CMT	0.790	0	0.008	0.054
Baseline EIFL	0.698	0	0.008	0.075
Baseline BCVA	0.801	−0.204	0.528	**<0.001**

*P* < 0.05 was considered statistically significant; significant *P* values are highlighted in bold.

**Figure 2.**
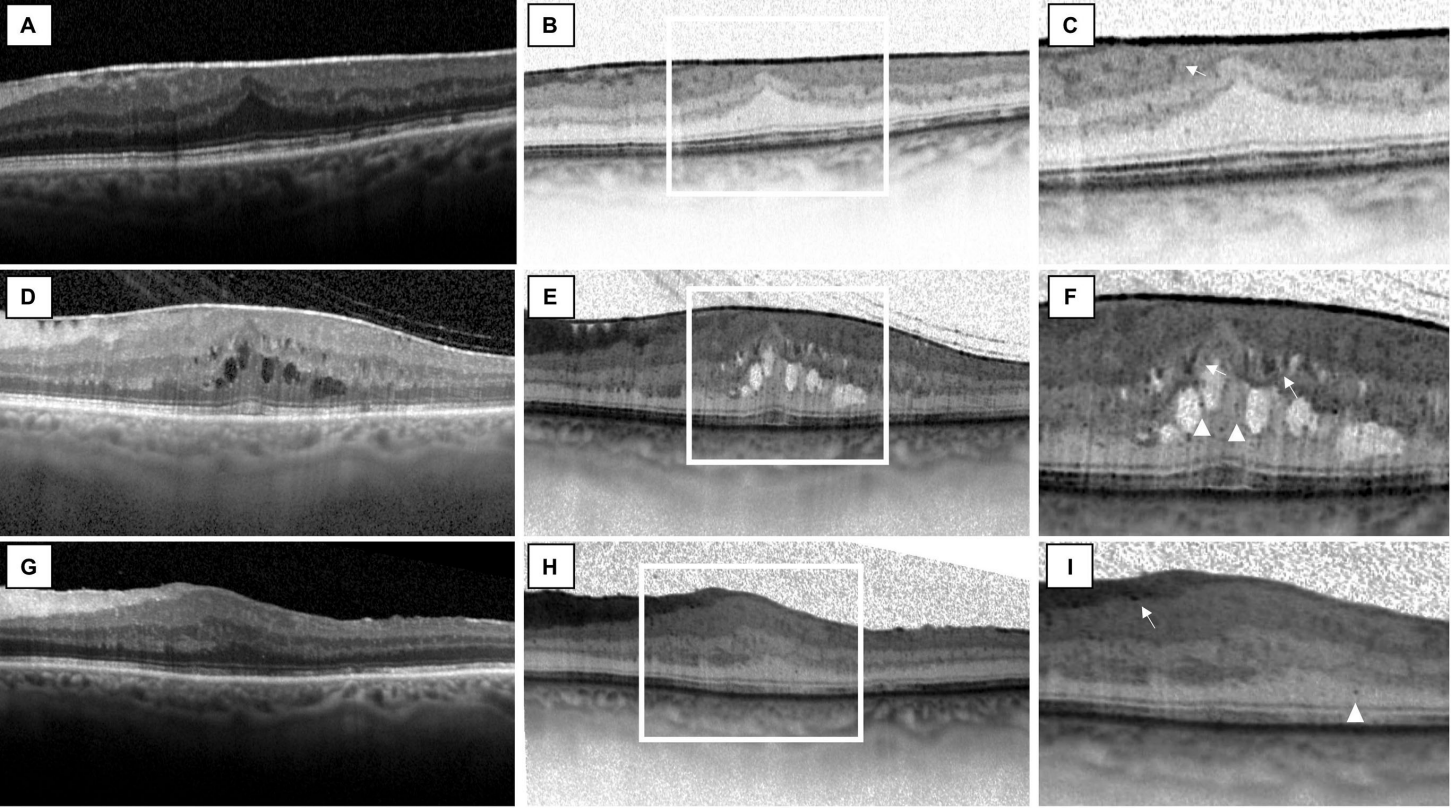
Progression of intraretinal HF in iERM. (**A**) OCT horizontal B-scan in a 68-year-old female patient with stage 3 iERM in her left eye. (**B**) Black-and-white mode of the same B-scan. (**C**) Magnification of the fovea is shown; note the presence of few HF in the EIFL thickness (*white arrow*). (**D**, **E**) OCT scan of the same eye after 16 months. Note the presence of cystoid macular edema, the increase in the EIFL thickness, and the increase in the number of HF in the EIFL and ONL. (**F**) Magnification of the fovea is shown. Note the presence of numerous HF in the EIFL thickness, which are consistently increased compared to the previous examination. Also, note the presence of HF in the ONL (*white triangle*). (**G**–**I**) OCT scan of the same eye 7 months after surgical peeling. Note the resolution of the cystoid macular edema and a consistent reduction in the number of HF in the EIFL and in the ONL in the central 1500 µm.

**Figure 3.**
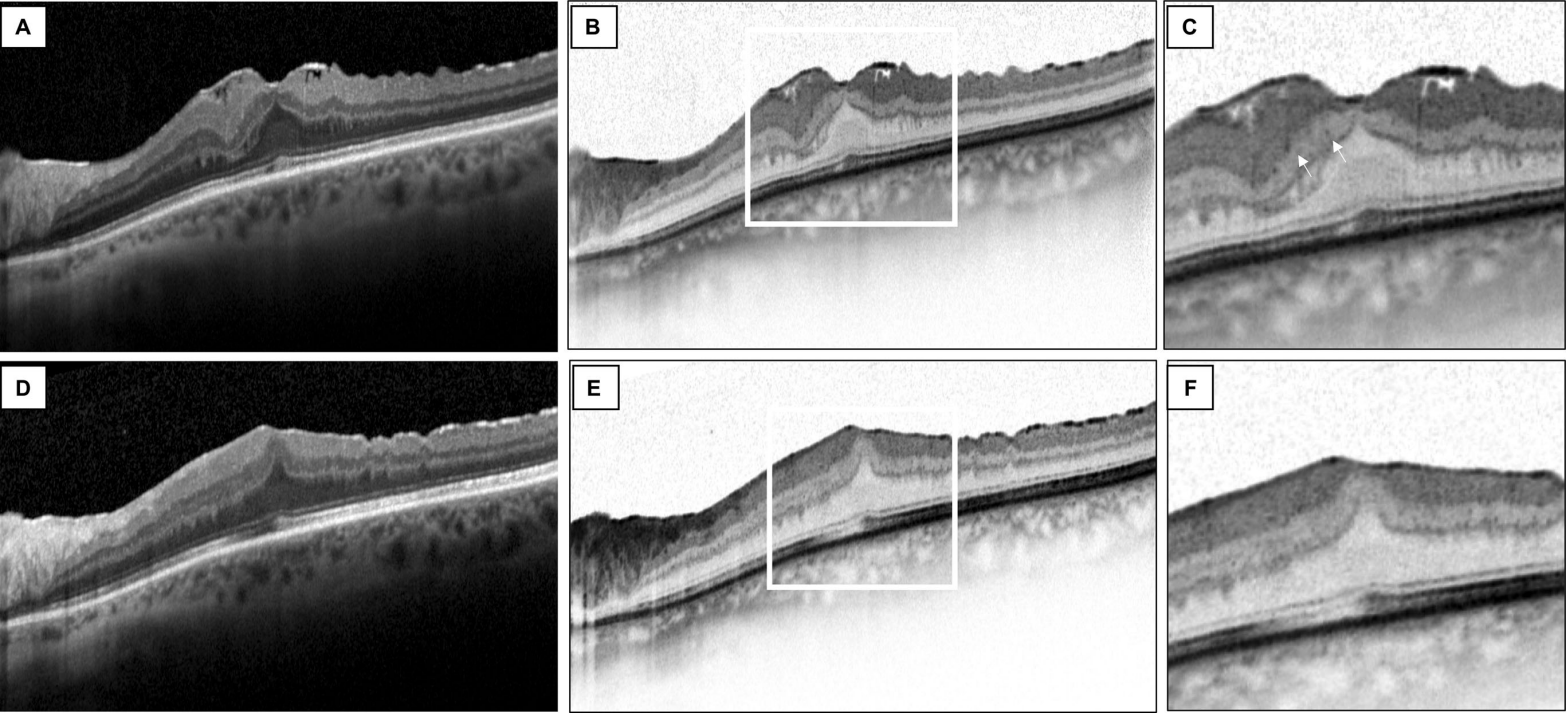
HF in stage 3 iERM. (**A**, **B**) OCT horizontal B-scan in a 65-year-old female patient with stage 3 iERM in the left eye. Note the presence of a thin EIFL and the presence of the central bouquet alteration. (**C**) Magnification of the fovea is shown; note the presence of few HF in the EIFL thickness (*white arrow*). (**D**, **E**) OCT scan of the same eye 7 months after surgical peeling. Note the persistence of the EIFL, central bouquet alteration, and disruption of the foveal EZ. (**F**) Note the absence of HF in the central 1500 µm.

Eyes with persistent HF in EIFL after surgery had a significantly higher EIFL thickness compared to eyes without HF (143.0 ± 60.37 vs. 103.68 ± 57.33, respectively; *P* = 0.036). HF count at the postoperative visit did not show any statistically significant correlation with BCVA at 6 months (r = 0.138, *P* = 0.451), SFCT (r = 0.010, *P* = 0.954), CMT (r = 0.154, *P* = 0.326), or EIFL thickness (r = 0.198, *P* = 0.203) (Figs. 2, 3; Table 5).

### HF and Visual Recovery

In the multivariate linear regression analysis evaluating predictors of postoperative BCVA, the presence of HF in the EIFL at baseline (β = 0.373, *P* = 0.048), age (β = 0.610, *P* = 0.015), and baseline BCVA (β = 0.801, *P* < 0.001) were significantly associated with worse postoperative BCVA. No other variables were significantly associated with the outcome (*P* > 0.05; Table 6).

## Discussion

In this study, we identified the presence of HF in the EIFL thickness in 74.5% of eyes with advanced iERM. These HF correlated with EIFL thickness at baseline, and they were observed in nearly 60% of cases after surgery, where they were associated with increased EIFL thickness following the surgical procedure. Furthermore, the presence of HF at baseline and after surgery was more common in stage 4 than in stage 3 iERM.

EIFL is a pathological retinal tissue observed in advanced stages of ERM that correlates with poorer visual acuity both before and after surgery.^13^^,^^14^ The exact mechanism behind EIFL formation remains unclear; however, chronic anteroposterior and centripetal traction from the ERM is thought to cause rearrangement and displacement of the inner retinal layers, resulting in the development of a continuous layer of inner and middle retinal tissues across the central fovea.^10^^,^^13^ Also, several studies have suggested that this traction can activate Müller cells, stimulating cell proliferation and gliosis.^13^^,^^20^^,^^21^ HF have been identified in various retinal conditions, where they serve as both clinical and prognostic biomarkers.^15^ It is believed that they represent aggregates of activated resident retinal microglial cells, playing a pivotal role in retinal inflammation.^22^ In a previous study, Chatziralli et al.^23^ observed the presence of HF in iERM in 13% of eyes; however, they did not specify whether the HF were located within the EIFL or ONL, nor did they correlate this finding with EIFL thickness or iERM grade. In a recent study, Zhao et al.^17^ identified the presence of HF at the vitreous–retinal interface using en face OCT of iERMs, proposing that these could be inflammatory cells involved in the pathogenesis of ERM formation. Notably, they found that these HF were present at all stages, with their numbers increasing as iERM grades became more severe. In the present study, we observed and characterized HF within the EIFL thickness in advanced iERM using SD-OCT, and we identified a correlation between the number of HF and EIFL thickness at baseline. Our findings support the hypothesis that HF in the EIFL thickness may represent activated microglial cells and that they could be involved in the pathogenesis of EIFL formation in advanced iERM.

At a mean follow-up of 7 months after surgery, HF in the EIFL were observed in 28 eyes (59.8%). Among eyes with HF at baseline, we observed a complete resolution of HF after surgery in seven eyes (20%), reduction in six eyes (17.1%), and persistence of the HF in 22 eyes (62.8%). Also, eyes with persistent HF after surgery had significantly higher EIFL thickness compared to those without HF, and persistent HF after surgery were more often encountered in stage 4 iERM. Although EIFL thickness may decrease significantly following surgery, it often persists for several months postoperatively. This prolonged change may result from alterations in Müller cells, along with the upregulation of glial intermediate filaments and glial fibrillary acidic protein (GFAP), which can increase tissue rigidity and impair its capacity for adaptive remodeling.^21^ Furthermore, Müller cells subjected to centripetal ERM traction may lose their structural compliance, thereby hindering the ability of the tissue to restore the normal foveal contour. Additionally, the surgical peeling process may induce further mechanical trauma, which could activate Müller cells. These activated Müller cells may then modulate immune and inflammatory responses by secreting proinflammatory cytokines, potentially leading to the activation of microglial cells. Our results support the hypothesis that HF may persist postoperatively. Further studies with longer follow-up are required to determine whether the reduction in ectopic layers after surgery is associated with a decrease in the presence of HF within the EIFL.

Another result of the present study was that the presence of HF within the EIFL at baseline, along with older age and worse baseline visual acuity, was significantly associated with poorer postoperative BCVA. Prior research has consistently shown that preoperative BCVA is the most reliable predictor of visual outcomes following iERM surgery.^24^ Chatziralli et al.^23^ found no association between HF and postoperative visual outcomes; however, their analysis did not differentiate the anatomical location of HF within retinal layers, which may explain the divergent results. Based on our findings, the location of HF appears to have different clinical implications. Specifically, HF located in the ONL were significantly associated with the presence of intraretinal cysts both pre- and postoperatively, suggesting that HF in the ONL may be related to fluid accumulation. However, HF in the ONL were not associated with EIFL thickness or visual outcomes, indicating a limited role in long-term visual prognosis. On the other hand, HF located within the EIFL were not associated with intraretinal cysts but were independently associated with worse postoperative BCVA. These findings suggest that HF in the EIFL may reflect more chronic or structural inner retinal changes, potentially related to glial proliferation or Müller cell activation, which could impact visual recovery. In conclusion, these findings suggest that HF within the EIFL could potentially serve as a novel imaging biomarker of retinal remodeling and could potentially contribute to preoperative risk stratification of visual outcomes in advanced iERM.

The main limitation of this study is the small patient cohort. Additionally, the retrospective design may have introduced bias. The follow-up period was also limited, and data from longer follow-ups were not included. Another limitation is that measurements were taken manually. Therefore, further studies with extended follow-up and the application of artificial intelligence software for automated analysis of OCT biomarkers are necessary to confirm our findings. In parallel, additional research is required to better understand the functional significance of HFs and to determine their predictive value regarding postoperative prognosis. Furthermore, additional studies are needed to include in the analysis the presence of HF in the vitreoretinal interface and choroid in advanced iERM, in order to gain a deeper understanding of their role in the pathophysiology and their potential impact on visual outcomes.

## Conclusions

We observed a significant association between HF in EIFL and both increased CMT and EIFL thickness prior to surgery. Furthermore, nearly 60% of the eyes exhibited HF in the EIFL thickness after surgery. In cases where HF persisted postoperatively, they were linked to an increase in postsurgical EIFL thickness. Also, their presence at baseline was associated with worse postoperative visual acuity. These findings suggest that HF in EIFL may represent microglial cells directly involved in the pathogenesis of EIFL. Additional studies, including histopathological analysis of the tissue, are necessary to fully understand the underlying nature of this novel OCT finding in advanced iERM.
